# Tackling diabetes as a team: co-designing healthcare interventions to engage couples living with type 1 diabetes

**DOI:** 10.1007/s00592-022-01900-4

**Published:** 2022-05-27

**Authors:** Rossella Messina, Emma Berry, Davide Golinelli, Sara Donetto, Chiara Reno, Simona Moscatiello, Gilberto Laffi, Jackie Sturt

**Affiliations:** 1 Department of Biomedical and Neuromotor Sciences, Alma Mater Studiorum-University of Bologna, Via San Giacomo, 12, 40126 Bologna, Italy; 2 School of Psychology, Queen’s University Belfast, Belfast, Ireland; 3 Florence Nightingale Faculty of Nursing, Midwifery and Palliative Care, King’s College London, London, UK; 4 Unit of Endocrinology and Prevention and Care of Diabetes, Sant’Orsola-Malpighi Hospital, Bologna, Italy

**Keywords:** Type 1 diabetes, Couples, Relationships, Emotional support, Qualitative study, Co-design

## Abstract

**Aims:**

Couples living with Type 1 diabetes: co-designing interventions to support them.

**Methods:**

This is a qualitative study comprising two phases. Phase I represents the exploratory phase, consisting of semi-structured interviews with persons with Type 1 diabetes (*N* = 16) and partners (*N* = 6). In the second phase, co-design principles guided workshops with healthcare professionals, persons with Type 1 diabetes, and partners (*N* = 7) to facilitate discussion of the key themes identified and solutions to engage couples living with Type 1 diabetes in diabetes care.

**Result:**

The key themes identified from phase I as priorities to target in future interventions were: (i) Emotional impact of diabetes and (ii) Partners’ involvement. Priority (i) captures the impact the emotional burden of diabetes management produces within couples’ relationship. Priority (ii) captures the request from partners to be more involved in diabetes management. Characteristics of the interventions suggested during the co-design phase II focused on engaging patients and partners via a counseling point in healthcare settings and tailored help for couples’ psychological support needs.

**Conclusions:**

Couples value pro-active intervention and support from their diabetes team or primary care for both the partners to live well with Type 1 diabetes.

**Supplementary Information:**

The online version contains supplementary material available at 10.1007/s00592-022-01900-4.

## Introduction

The psychological impact of Type 1 diabetes extends beyond the individual, placing strain on relationships and requiring considerable adaptation and resilience at the familial level [1–4]. Among couples living with chronic physical conditions, healthy psychological adaptation is shaped by factors such as quality of communication, cohesion and support [3, 4], and these factors influence Type 1 diabetes self-management too [5]. As with many chronic health conditions, living with diabetes requires a holistic package of care: medical treatment, education, and social and emotional support [6]. While medical advances are vast and educational resources have become more refined, adequate psychological support, and particularly support that considers the wider family context, remains scarce.

Tackling Type 1 diabetes is a team effort, and evidence suggests that people with chronic health conditions achieve better health outcomes when they live in a couple [7]. Equally, if support provided by partners is inappropriate or unhelpful (e.g., over or under-protective) this can exacerbate the self-management and emotional challenges experienced by people with Type 1 diabetes [5]. Partners and spouses of people living with diabetes often worry about complications and hypoglycaemia and feel uncertain about how to help their loved one with diabetes [8–10]. Such emotional concerns can manifest in the way that partners/spouses support or communicate with their loved one, which highlights the need to consider the role of partners in diabetes interventions.

While it is important that partners and spouses are considered in interventions to support psychosocial wellbeing in contexts where couples are struggling to cope, systematic reviews have identified a dearth of interventions for couples living with Type 1 diabetes [5, 11]. Research suggests that psychosocial adjustment among couples living with chronic conditions such as diabetes can be supported by interventions which focus on strengthening communication, on encouraging dyadic coping, and on actively involving couples in the developmental process [5, 11–13]. To date, possibly due to a lack of clarity on what kind of support would be most helpful for couples living with Type 1 diabetes, few interventions have been developed to specifically address the unique struggles these couples face [14]. Given this knowledge gap, this study explores the perspectives of people with Type 1 diabetes, partners, and healthcare professionals with a view to informing the development of tailored support for couples living with Type 1 diabetes.

In this paper we explore the challenges and support needs of couples living with Type 1 diabetes in Italy and the co-design of solutions to enhance couples’ well-being and diabetes self-management. We refer to three groups of participants involved in two distinct and consecutive phases: an exploratory phase I involving interviews with persons with Type 1 diabetes (PWD) and partners of people with Type 1 diabetes (T1D partners) and a co-design phase II involving a joint workshop for PWD, T1D partners, and healthcare professionals (HCP).

## Methods

### Design

This qualitative study consisted of two developmental phases.

Phase I represents the exploratory stage where psychological, interpersonal, and practical needs and challenges experienced by PWDs and T1D partners were explored via individual semi-structured interviews.

Findings from the interviews informed the development of a series of illustrations representing common experiences/issues identified from our thematic analysis of the transcripts. Phase II was the co-design stage (see below for more detail). Experiences/issues identified in phase 1 were further discussed in this phase in a group composed by PWDs, T1D partners, and healthcare professionals to identify characteristics and potential solutions to be implemented in healthcare services to support people with diabetes and their partners.

In this phase of the study, following a photo-elicitation method [15], the illustrations were used to facilitate/prompt the discussion within the group. In fact, images can evoke emphatic understanding of how other people experience their world.

### Co-Design approach

Participatory design has been used as a method for involving patients or end-users in the design of health services in order to develop interventions that are more likely to be engaging, relevant, and effective. The current study used a modified version of the Experience Based Co-Design (EBCD) [16, 17] approach to address the research aims. EBCD is an approach, developed in England in 2005/2006, for quality improvement of healthcare services. Through a co-design process the approach entails staff, patients and carers reflecting on their experiences of a service, working together to identify improvement priorities, devising and implementing changes, and then jointly reflecting on their achievements [16, 17]. The EBCD approach is traditionally organized into six stages [16, 17]: setting up, engaging staff and gathering experiences, engaging patients and gathering experiences, co-design meeting with a visual prompt consisting of an edited film from patients’ experiences, small co-design teams, final celebration event.

Our modified version included the following phases: setting up, engaging patients and partners with interviews, and a co- design joint meeting with PWDs, T1D partners and HCPs and mixed smaller co-design teams. To facilitate the discussion, we replaced the edited film with illustrations portraying the experiences of couples living with Type 1 diabetes. The illustrations were created ad-hoc by the Illustrator Emily Monteverde for the study and were borrowed with permission from a Danish study [18].

### Participants

In Phase I, people with diabetes (PWD, *N* = 16) and partners with no diabetes (T1D partners, *N* = 6) participated in the interviews. The co-design Phase II was joined by HCPs and in particular nurses, healthcare assistants and physicians (*N* = 5), PWD (*N* = 1) and T1D partners (*N* = 1). PWDs and T1D partners were not required to be part of the same couple; rather, they could be independent of each other. PWD and partners were therefore recruited individually and so inclusion criteria were established for PWDs and for T1D partners.

Inclusion criteria for PWDs were as follows: adults aged 18–80 years diagnosed with Type 1 diabetes at least 6 months prior to taking part in the study, co-habiting with a spouse or partner for at least 1 year.

Inclusion criteria for T1D partners were as follows: adults aged 18–80 years, who had been cohabiting with a spouse or a partner with Type 1 diabetes (diagnosed at least 6 months previously) for at least 1 year.

Participants were excluded if they had any major impairments that might impede study completion (e.g., dementia) and clinical diagnosis of Type 2 diabetes, gestational or iatrogenic diabetes. Not being in a romantic relationship and not cohabiting was also an exclusion criterion.

PWD and T1D partners were recruited from the Endocrinology and prevention and care of diabetes Unit of Sant’Orsola- Malpighi Hospital Bologna and through the online platforms (Facebook pages, e-mails networks and websites) of the three Diabetes charities, collaborating with the research group. Recruitment took place between February and June 2019. PWDs and T1D partners who expressed the opportunity to participate to the study, were offered the possibility to choose which of the two phases take part. HCPs who agreed to participate to phase II were part of the clinical team of the same Unit.

### Data collection

#### Phase I: semi-structured interviews

Individual semi-structured interviews were conducted with PWD and T1D partners [19, 20]. The interviews lasted 15-45 minutes. Draft interview guides were informed by relevant systematic reviews [5, 10] and by the aims of the study. They focused on couples’ experiences of living with T1D, with special attention given to how these experiences may have impacted their relationship and diabetes management. The draft interview guides were refined on the basis of the feedback from a study advisory group consisting of a diabetologist, two people with diabetes and a representative of a local diabetes charity (see final approved version in Appendix 1). The interviews were carried out in a private room in the Endocrinology and prevention and care of diabetes Unit or via telephone or Skype by a psychologist (RM) with expertise in research and clinical practice in diabetes care. Demographic (T1D partners and PWD) and clinical data (PWD) were collected at the beginning of the interview. Interviews were conducted in Italian and were digitally recorded and transcribed verbatim by the first author (RM).

#### Phase II: co-design workshop

Diabetes health care professionals and newly recruited PWD and T1D partners were invited to participate in a three- hour workshop to identify priorities and suggest solutions to be developed in clinical practice. During the workshop, the photo-elicitation method was used to facilitate the discussion. The co-design workshop was organized around three key stages. In stage 1, workshop facilitators explained the principles of the co-design approach to healthcare improvements and introduced the findings from analysis of the interviews; stage 2 involved HCPs and two representatives of PWD and T1D partners to discuss areas for improvement with the help of illustrations; in the third and final stage participants worked in mixed groups to outline potential improvements to existing services on the priorities discussed.

In stage 3, two distinct and mixed working groups were created. Group A consisted of: 1 T1D partner, 1 nurse, 1 diabetologist and 1 diabetes educator. Group B consisted of: 1 PWD, 1 diabetologist and 1 diabetes educator.

The two groups were invited to: identify 2 priorities, indicate characteristics for quality improvement, and produce a poster as a final report with solutions and characteristics identified across each priority. Notes taken by RM during these presentations were used in the preparation of the report. Photos from the three key stages are reported in Appendix 2. The workshop was facilitated by two of the authors (SD, RM).

### Qualitative analysis

NVivo 12 Plus software was used to organize, store and code interview transcripts.

Data were analyzed using thematic analysis, applying an inductive coding technique to detecting the key themes. Analysis followed six phases [21]: transcription and familiarization of the data; preliminary coding; collection of codes and identification of themes; review of themes; confirming and defining themes; and reporting key themes. The researcher coordinating the study (RM) maintained reflexivity [22] by keeping a diary to record methodological and analytic process.

RM and DG held regular research meetings to discuss codes and examine any serious discrepancies in interpretation of data. Key themes were then discussed with co-author SD in preparation for the co-design workshop and confirmed and re- defined for the final report by another co-author (JS). The Consolidated criteria for Reporting Qualitative research (COREQ) check-list have been used to inform this final report [23].

## Results

### Phase I

Sixteen PWD and six T1D partners were interviewed by the first author (RM) in phase 1. Of these, three were couples; however, all participants were interviewed individually to avoid eliciting conflictual dynamics that we would not be able to support within the clinical unit during the interview session. Socio-demographics for interview (phase 1) and workshop (phase 2) participants are summarized in Table 1.

**Table 1:** Characteristics of participants interviewed (PWD and T1D partners, *N* = 22) and attended the workshop (HCPs, PWD and T1D partners, *N *= 7) (*N *= 29 in total)

Interviews participants	PWD	T1D partners
Female, *n*	9	2
Male, *n*	7	4
*Age range, n*		
20s	0	1
30s	1	0
40s	2	4
50s	9	0
60s	4	1
Relationship duration*		
0–5 years	1	2
> 5–10 years	0	1
> 10 years	15	3
*Time since diagnosis*		
< 1 year	2	1
1–15 years	3	2
> 15 years	11	3

Workshop participants	*n*	
*Diabetes health care professionals*		
Consultant diabetologist	2	
Diabetes specialist nurse	1	
Diabetes educators	2	
PWD	1	
T1D partners	1	

*PWD*: person with diabetes,* T1D partners*: partners of people with a diagnosis of diabetes,* HCPs*: healthcare professionals

The themes identified related to: (i) different perspectives and needs related to living with diabetes, (ii) diabetes management and its impact on couples’ relationships, (iii) diabetes distress experienced by partners, and (iv) hypoglycemia management and its emotional effect on both the partners.

Through these themes, we identified the following priorities: “Diabetes emotional impact” and “Partners’ involvement”.

### Diabetes emotional impact

This priority describes how emotional burden related to diabetes management impacts couples’ memberships. T1D partners felt frustrated for two reasons: they did not know how to best support PWDs and at the same time, their efforts are not recognized by PWDs. A partner said [T1D partner #7, male]: “it’s like no one is noticing that diabetes is having an effect also on me!”.

In relation to hypoglycaemia management, partners are often able to read the signs and symptoms that indicate an impending hypoglycemic crisis: “…during the night I heard her breath that was going to change the intensity, I made her wake up and said are you ok? This happened frequently before she changed her medications'' [T1D partner #2, male].

Most of the T1D partners participating in the study reported that they experience a sense of emotional burden as they cope with worries about hypoglycaemia, fear of long-term complications, battles with their PWD over blood glucose monitoring, and other self-care behaviors.

On the other hand, many PWDs in the study felt frustrated by their partners’ continuous apprehensive emotional responses: “I always try to do everything by myself […] until he realizes that I am in hypoglycemia and starts to worry. I feel bad afterwards. Not for my blood sugar, but I feel morally sick because it's like I'm not an independent person and this thing bothers me extremely. He becomes anxious and automatically transmits this to me, and therefore I not only have to try to get my blood sugar raised, but I have to try to lower the anxiety that he has transmitted to me” [PWD#8, female].

Nonetheless, both PWDs and T1D partners recognize the need for couples’ relationship support, particularly when their emotional responses around diabetes management interferes with their quality of life as a couple. For instance, T1D partner #7 (male) said: “communicating our emotions [with the PWD] is very important but sometimes is not allowed by the partner. Because of this, it becomes difficult to interact with those who have diabetes. A discussion, a criticism or an offense becomes something that is no longer a constructive dialogue, but it distances me further. I am forced to stay out of her diabetes stuffs”. Similarly, PWD#4 (female) reported: “Particularly if the diagnosis occurs during the relationship, for the person, and therefore for the couple, it is a trauma. For this reason, having a space for processing this and for support […] would be useful”.

### Partners’ involvement

This priority conveys the request from partners of being involved in the management of diabetes and the negotiation of it with PWDs as well as considering their point of view.

Type 1 diabetes, as a chronic condition, is part of a person's life "like a backpack" [PWD #12, female] and thus it inevitably influences the couple's relationship. T1D partners expressed the need to receive information on diabetes management and how to best support the other person with their treatment; however, this can be experienced as intrusive by the PWD, and therefore the support needs to be negotiated.

T1D partner #5 (female) highlighted how she was heavily involved in her partner’s daily management of diabetes. When asked about the kind of support she would find helpful as the partner of a person with Type-1 diabetes, she suggested that she would appreciate it if her husband took more responsibility for his diabetes management by learning carbohydrate counting: “I would like him (PWD) to take a course, for example the one on carbohydrate counting. This would solve the problem he has, because he would learn how to manage meals or outings”.

A participating person with Type-1 diabetes suggested that couple counselling may be a fruitful way to achieve a shared perspective on the condition, in particular one that does not emphasize its disabling aspects. They said: “I think it would be useful to do couple counseling. He is always on at me because of my disease or thinks that I am very sick. So, it would be better to manage diabetes not as a disabling disease (even if it is), but by trying to make life lighter, not even heavier than it already is. I would recommend it to everyone” (PWD #8, female). This PWD emphasized the need to include partners in diabetes care because this may be of help to approach, accept, and cope with and integrate diabetes as part of their lives as a couple.

This argument can be controversial within couples. Some PWDs express the need to interpose boundaries between themselves, their partners, and their own diabetes. A PWD (#16, female) said “Nobody else who doesn’t have diabetes may ever know what this means and how to manage it”, suggesting that no learning or education would provide enough understanding of the lived experience of the condition. PWDs in our study also expressed their fear of being judged by their partners on how they cope with and manage their diabetes. They recognized the support of their partners with impending episodes of hypoglycemia, but even in those cases, they saw their partners’ involvement as a variable that should still be negotiated.

T1D partners expressed the need of being recognized by PWDs and HCPs in their role as allies. According to them, diabetes could be a common struggle rather than an individual affair: “in my opinion it [being recognized by PWDs and HCPs in their role as allies] could be helpful because in any case the disease is experienced by both, and it tends to divide rather than unite. […] So afterwards it becomes a double effort within the couple trying to stay together. Instead, it could be a reason to fight for a common struggle. There is no involvement [by the HCPs], which in my opinion should be crucial […] I don't understand why we are not involved” [T1D partner #7, male]. These tensions between partners on how to be involved in diabetes management may lead to issues in the relationship related to T1D partners trying to understand and define their role vis a vis their partner's diabetes. The partners even suggested that in certain circumstances partners could be contacted by healthcare professionals directly for a more in-depth understanding of the impact of diabetes management on the couple.

### Phase II

Five HCPs, one T1D partner representative, and one PWD representative participated in the co-design workshop in phase 2. Seven participants in total took part in the co-design workshop. While HCPs expressed willingness to contribute to this workshop, it was more challenging to recruit PWDs and T1D partners. This may have been due to a variety of reasons. It may be that the interactive nature of the methodology used in this phase meant people with diabetes and their partners were unwilling to share personal experiences in the company of HCPs. Or it may be that there is little awareness, both in the community and in the context of healthcare services, of the relevance of this topic, which may have translated into poor participation in the study. Once the priorities were selected in phase I, the two mixed groups (A and B, see methods) identified practical solutions for the key themes earlier identified.

The final outputs from the two mixed groups suggest that both groups recognize the need to involve T1D partners in the diabetes healthcare pathway. Both groups came up with a pragmatic and feasible solution: to facilitate a counseling point for couples or for PWD and T1D partners individually, supported by the HCPs of the diabetes center and a psychologist focused on the relationships. This intervention could be face-to-face or via telephone/teleconferencing, depending on the needs of the couple, and would aim to provide interpersonal support for the couple relationship as well as dyadic diabetes management support.

The following suggestions were also expressed by the two groups, as premises of the development of the intervention:

(1) there is a need to introduce T1D partners in diabetes care because they could be allies in diabetes management, but the involvement needs to be negotiated with PWDs; (2) there is a need for educational support, including information on hypoglycaemia management, technological devices, and carbohydrate counting; (3) there is a need for emotional support for T1D partners and for the couple, which may include support from psychologists within the multidisciplinary team; (4) there is a need for diabetes charities and healthcare services to work together to support the planning, delivery, and advertisement of such an intervention as it may be delivered as part of routine clinics and/or in community settings; (5) interventions could be delivered to individuals and/or to groups to meet the preferences of people with diabetes and individual healthcare teams.

During mixed groups stage of the meeting, HCP representatives were asked to discuss the feasibility of integrating these interventions in routine clinical practice and to this proposal an HCP said: “The intervention is doable and spaces, human and financial resources are available. We just need a change of perspectives in healthcare policies on introducing partners and families in adult diabetes care”.

## Discussion

The aim of this qualitative study was to explore the challenges and support needs of couples living with Type 1 diabetes in Italy and co-design solutions to enhance couples’ well-being and diabetes self-management. To date few interventions have been developed and evaluated to specifically address the needs of couples who are struggling to cope with diabetes self-management despite evidence that it is a considerable challenge.

The current study identified that ‘Diabetes emotional impact’ and ‘Partners’ involvement’ are key aspects to consider when designing interventions to support couples living with Type 1 diabetes.

Diabetes emotional impact, which can be defined as the emotional responses to diabetes itself as a chronic condition and as the burden related to diabetes self-management, has been identified in T1D partners as well. It is known that people living with Type 1 diabetes may experience diabetes distress [24] and this distress is not experienced only by the person with diabetes but also their partner [25]. Similar to the findings by Polonsky and colleagues [25], our study shows that diabetes management usually involves the partners in practical activities, which may cause an emotional distress within the couple. Approximately 30% of patients with diabetes can experience diabetes distress. Diabetes distress is a multifaceted construct, which, in the context of Type 1 diabetes, encompasses psychological struggles related to healthcare providers and family/partners, perceived social appraisals, concerns about hypoglycemia, feeling powerless, and distress associated with self-care and eating behavior [26]. Partners of people with Type 1 diabetes can also experience diabetes distress, which typically manifests as feelings of uncertainty as to how best to help their significant other manage their diabetes and experiences of frustration and loneliness when their own emotional struggles go unnoticed or are neglected [25].

T1D partners’ involvement can be defined as the request from partners of collaborating in diabetes management, and, from PWD, the need of negotiating the support, to be experienced as less intrusive as possible.

Our study suggests that both emotional impact of diabetes and involvement in diabetes management have an effect on hypoglycemia management, diabetes self-management activities, and diabetes distress experienced by PWD and T1D partners. Further studies need to investigate whether diabetes emotional responses and the different ways of approaching diabetes between T1D partners and between couples depends on level of diabetes acceptance, attitudes towards living with a chronic condition, attributed meanings towards diabetes, or other aspects of individual and couples psychological wellbeing. Exploring these complex psychosocial processes and experiences may help to create personalized interventions that focus on individuals and couples’ strengths and areas in need of support.

The co-design approach adopted in this study enabled us to take into account the perspectives of PWD, T1D partners, and healthcare professionals, but also enabled them to share perspectives in a group setting. Moreover, this methodology empowered couples living with diabetes and healthcare providers to inform the focus of the intervention (i.e., by identifying priority themes) and to co-design solutions to ensure that the intervention is centered on the couples and is designed to help, and to also involve the healthcare providers in the planning of this support.

The joint workshop identified priorities to support couples with communication and dyadic coping, to empower T1D partners in the diabetes self-care regime, and to alleviate the burden of diabetes for PWD. Of note, couples felt that a ‘listening point’ i.e., a supportive space to discuss challenges and work through solutions, would be an appropriate solution to help couples who are struggling to cope with diabetes and impact of diabetes on their relationship.

The role of the emotional and psychological support has been underlined from the healthcare professionals as well as PWD and T1D partners who participated in the workshop. Engagement of couples living with Type 1 diabetes in diabetes healthcare settings enable them to recognize and respect each other's needs and boundaries and negotiate support and involvement. This can influence diabetes distress, as the partner can be considered as an ally or a resource to better support the daily fatigue of having to live with Type 1 diabetes and the demands of accommodating the condition.

Many people with a diagnosis of Type 1 diabetes in adulthood can experience a disruption in their lives before and after the diagnosis [26], so emotional and educational support is needed. The partner is thus involved in the diabetes management and together they face challenges that are not often taken into account by healthcare pathways.

As suggested by Berry et al. [11], interventions for couple can be more effective than patient-only interventions or controls across various patient and partner outcomes. Couples interventions tend to favor a skills-based or a relationship-based approach, which strongly influences the types of outcomes effectively targeted. Supporting couples living with Type 1 diabetes means to support them emotionally as individuals but also as counterparts of a dyadic relationship. Moreover, this requires tailoring diabetes-related information and endorsing coping strategies, which facilitate healthier dyadic adjustment, thus supporting better choices and outcomes related to health.

Our qualitative study had some limitations. First, we recruited fewer PWD and partners for the workshop than for the first phase of the study. This means that the findings from phase II draw primarily on HCPs’ perspectives and therefore we cannot assume that the findings drawn from this phase represents the voices of PWD and partners with different experiences and backgrounds. Second, our participant sample captures the perspectives of middle-aged couples and with medium and long-term relationships. No young couples and young age participants were included and thus our study does not represent these age groups and relationship types. Furthermore, we did not include sub-analysis based on the timing of diabetes diagnosis—i.e., adulthood versus childhood—due to sample size limitations. Further research could explore any differences related to age at diagnosis, given the unique adjustment challenges experienced with later diagnoses of Type 1 diabetes [27], where couples may also have established relationships *pre-diabetes*. Relatedly, the age range of PWDs and partners in the sample varies widely (participants are aged between 18 and 80 years). Although this was a deliberate effort to capture a variety of perspectives across different life phases, due to the lack of sub-analysis per age cohort, this may overlook important psychosocial priorities for younger versus older couples. For example, psychosocial priorities for young adult couples may focus on managing the threat posed by diabetes to achieving specific milestones (e.g., working toward a career) and sexual needs [28, 29]. Whereas, older adult couples may be more concerned about living with comorbidities [30] and fear of death [28]. It is important that psychosocial interventions for couples are tailored to the life stage of the couple, and future research should thus explore how the proposed intervention approach can be tailored to couples of different life stages.

In further developments of an intervention, it would be pertinent to explore who might deliver the intervention, to establish in which setting such an intervention can feasibly be delivered, to refine the intervention design, and to plan initial piloting and appropriate evaluation strategies. Given the nature of the intervention proposed, it is imperative for its effectiveness that intervention facilitators are equipped with the knowledge and experience to recognize how psychological and interpersonal processes can impact medical outcomes, that is, how challenging thoughts and feelings and relationship difficulties influence self-care. With this in mind, further research could consider this type of intervention requires a psychologist, upskilled diabetes specialist, or primary care provider. As for the service setting, delivery by healthcare providers outside of secondary care—i.e., hospital settings- can present a more accessible and cost- efficient service for families living with Type 1 diabetes, meaning a greater propensity for adoption in diabetes care pathways. It is also important to consider whether the delivery of interventions would be group-based, for dyads or hybrid. There are successful group-based experiences of self-management programs for people with chronic conditions and their caregivers [31]. In fact, a group-based format can facilitate greater social support [32, 33] by encouraging communication with partners and the disclosure of personal struggles in a supportive setting with people who face similar challenges.

## Conclusions

Type 1 diabetes management needs a team effort. In this study, we found that T1D partners and PWD expressed different perspectives on how to cope with Type 1 diabetes, but experienced the same emotional and educational needs. Priorities identified were diabetes emotional impact within the couple, and T1D partners’ need of being involved by PWD on diabetes management. HCPs reflected on the opportunity to introduce T1D partners in diabetes care via counseling points offering diabetes education and psychological support for couples or just for T1D partners. This study provides a sound foundation to establish an intervention based on the needs and solutions voiced by people with diabetes, their partners, and healthcare providers.

## Supplementary Information

Below is the link to the electronic supplementary material.

Supplementary file1 (DOCX 1463 KB)

## Data Availability

The data sets generated during and/or analyzed during this study are available from the corresponding author on reasonable request.
