# Supplementary material for: Tackling diabetes as a team: co-designing healthcare interventions to engage couples living with type 1 diabetes
Source: Acta Diabetol. 2022 May 27;59(8):1053–61. doi: 10.1007/s00592-022-01900-4 (PMC9242905; doi:10.1007/s00592-022-01900-4)

**Appendix 1.** Interviews’ schedule

Semi-structured interview **for PWDs**

1. What impact do you think diabetes has on your life during this period (eg. negative, positive or both)?

2. How does diabetes affect your relationship with your partner?

3. How did you get your partner involved in diabetes management?

4. What kind of support did you receive regarding your partner's involvement in diabetes? Was it useful or not?

5. What kind of support would you need to involve your partner in managing diabetes that meets your needs?

6. Do you think diabetes interferes with your vision of the future with your partner?

7. Did diabetes interfere with your intimate sexual relationships? If yes, has / have you received any support for this?

8. Has the use of new diabetes management technologies (eg. insulin pump) interfered in any way on your partner and your relationship?

9. What effect do you think hypo or hyperglycemia can have on your partner and your relationship?

Semi-structured interview **for T1D partners**

1. What is your experience of living with a partner who is diagnosed with type 1 diabetes?

2. How much does diabetes affect your relationship and how?

3. How involved is you in managing diabetes?

4. What type of support has / have you received that may have helped you and your partner manage diabetes as part of your relationship? Was it helpful or not?

5. What kind of support would you like to receive to help you manage the impact that diabetes has on your relationship?

6. Do you think diabetes affects your vision of the future with your partner? If so, how?

7. Did diabetes interfere with your intimate sexual relationships? If yes, has / have you received any support for this?

8. Has the use of new technologies for the management of diabetes (eg insulin pump) interfered in any way on you or your relationship?

9. What effect does your partner's hypo or hyperglycemia have on you and your relationship?

**Appendix 2**. Photos of the co-design workshop


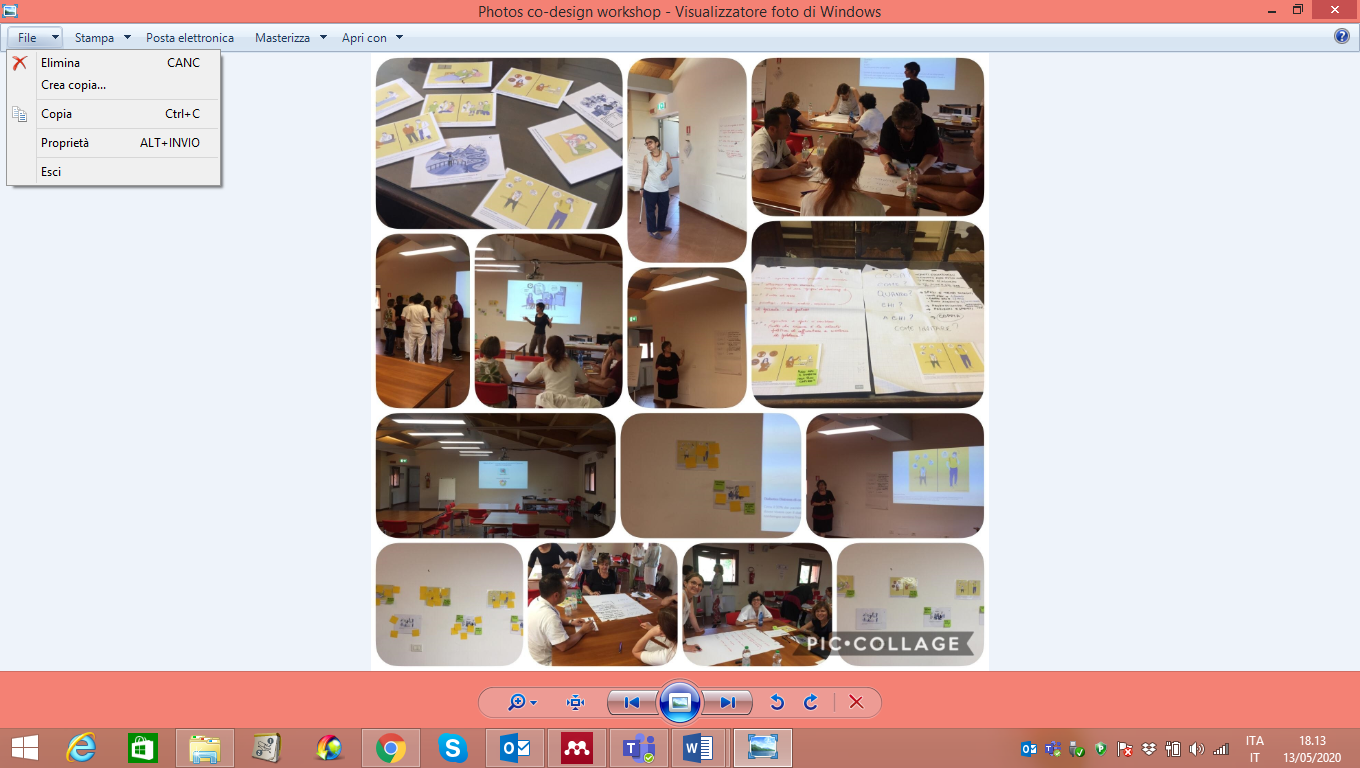


**Appendix 3.** Illustrations of priorities identified and representing: Diabetes emotional impact (A) and Partners’ involvement (B).

***A***


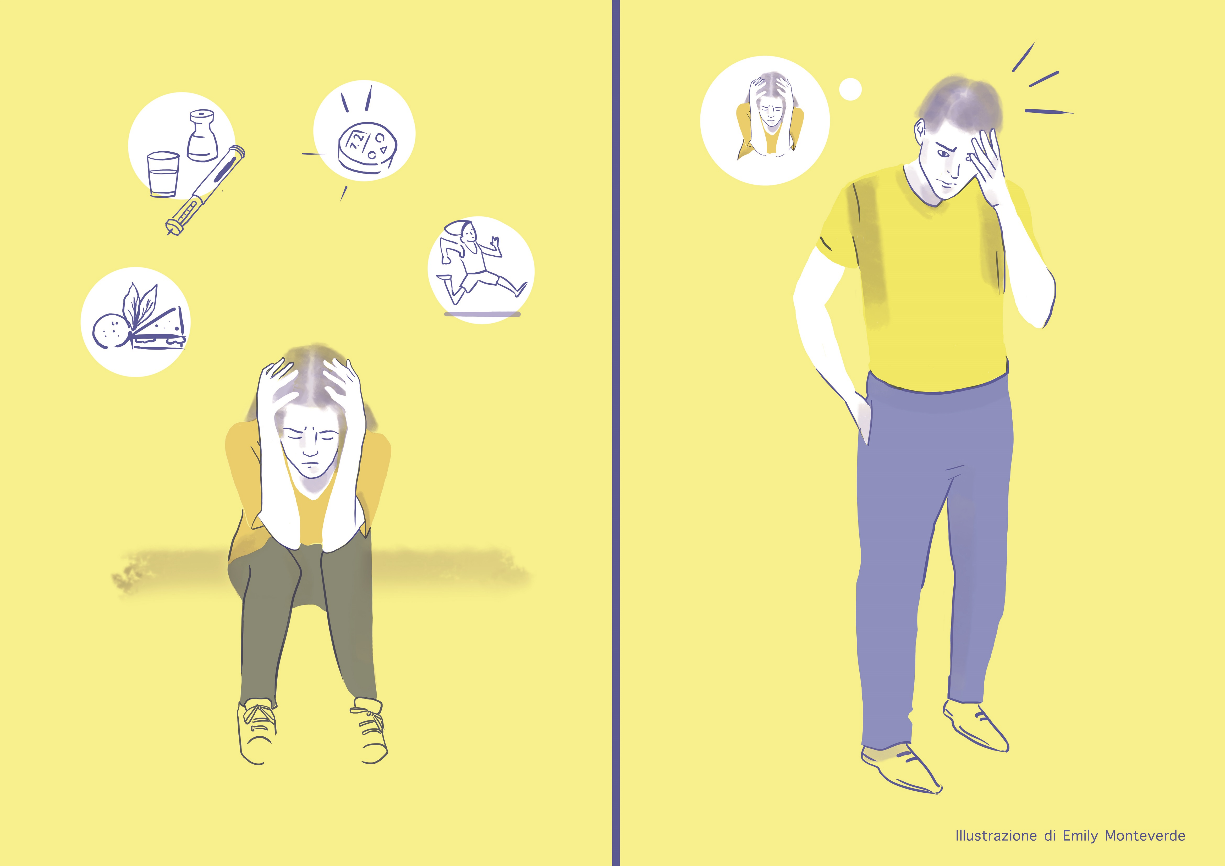


***B***


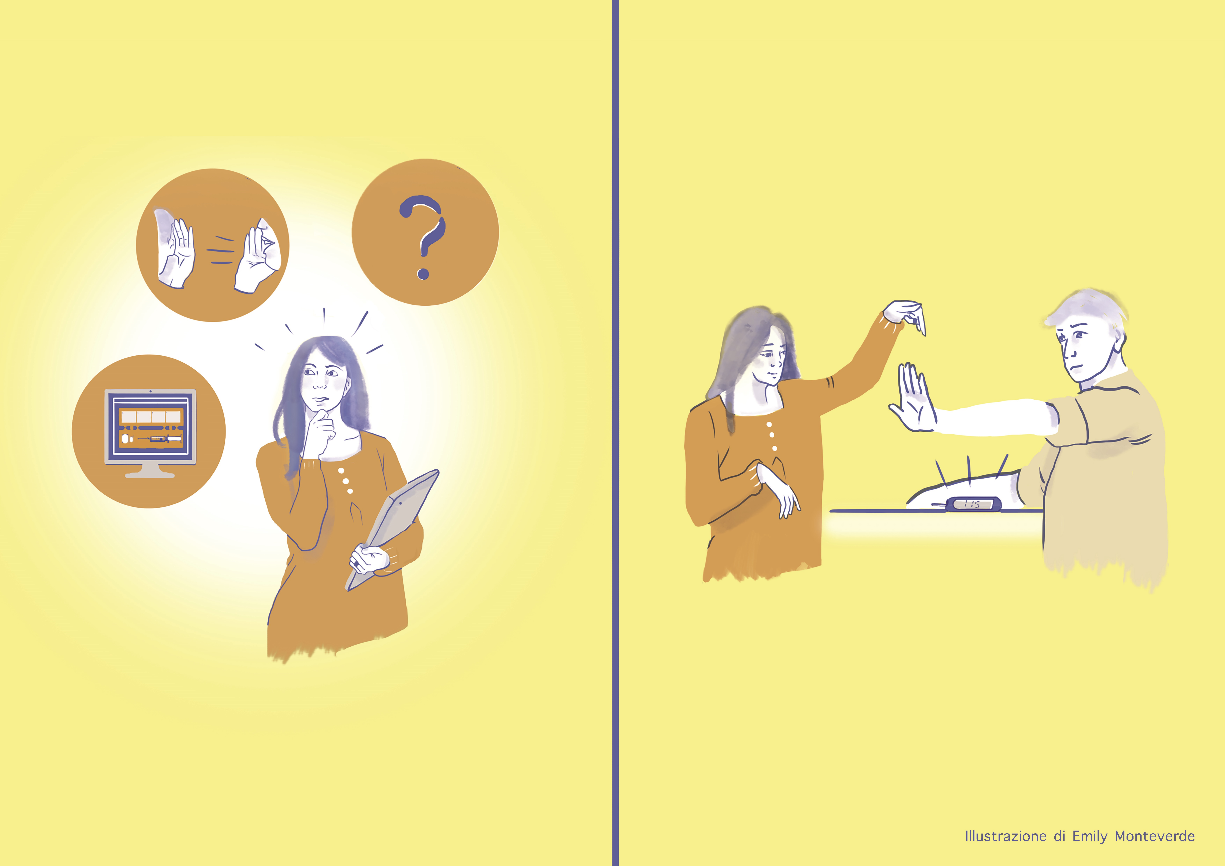

Supplement: Supplementary file 1 — Supplementary file1 (DOCX 1463 KB) [file 592_2022_1900_MOESM1_ESM.docx]
